# The Biosynthesis of 1-octene-3-ol by a Multifunctional Fatty Acid Dioxygenase and Hydroperoxide Lyase in *Agaricus bisporus*

**DOI:** 10.3390/jof8080827

**Published:** 2022-08-08

**Authors:** Tongfu Su, Yuannan Chen, Haohao Liu, Yuqian Gao, Jiawen Guo, Yanan Li, Yuancheng Qi, Liyou Qiu

**Affiliations:** Key Laboratory of Enzyme Engineering of Agricultural Microbiology, Ministry of Agriculture and Rural Affairs, College of Life Sciences, Henan Agricultural University, Zhengzhou 450002, China

**Keywords:** button mushroom, 1-octen-3-ol, linoleic acid, enzymatic pathway

## Abstract

The biosynthetic pathway from linoleic acid to 1-octen-3-ol in *Agaricus bisporus* has long been established, in which linoleic acid is converted to 10-hydroperoxide (10-HPOD) by deoxygenation, and 10-HPOD is subsequently cleaved to yield 1-octene-3-ol and 10-oxodecanoic acid. However, the corresponding enzymes have not been identified and cloned. In the present study, four putative genes involved in oxylipid biosynthesis, including one lipoxygenase gene named *AbLOX*, two linoleate diol synthase genes named *AbLDS*1 and *AbLDS*2, and one hydroperoxide lyase gene named *AbHPL* were retrieved from the *A. bisporus* genome by a homology search and cloned and expressed prokaryotically. *Ab*LOX, *Ab*LDS1, and *Ab*LDS2 all exhibited fatty acid dioxygenase activity, catalyzing the conversion of linoleic acid to generate hydroperoxide, and *Ab*HPL showed a cleaving hydroperoxide activity, as was determined by the KI-starch method. *Ab*LOX and *Ab*HPL catalyzed linoleic acid to 1-octen-3-ol with an optimum temperature of 35 °C and an optimum pH of 7.2, whereas *Ab*LDS1, *Ab*LDS2, and *Ab*HPL catalyzed linoleic acid without 1-octen-3-ol. Reduced *AbLOX* expression in antisense *AbLOX* transformants was correlated with a decrease in the yield of 1-octen-3-ol. *Ab*LOX and *Ab*HPL were highly homologous to the sesquiterpene synthase Cop4 of *Coprinus cinerea* and the yeast sterol C-22 desaturase, respectively. These results reveal that the enzymes for the oxidative cleavage of linoleic acid to synthesize 1-octen-3-ol in *A. bisporus* are the multifunctional fatty acid dioxygenase *Ab*LOX and hydroperoxide lyase *Ab*HPL.

## 1. Introduction

Briefly, 1-octen-3-ol is the source of a characteristic mushroom aroma first found in *Tricholoma matsutake* and is known as mushroom alcohol [1]. It was subsequently discovered to be in a variety of mushrooms and molds [2,3,4], plants, animals, and their products [5,6]. The main aromatic component of the button mushroom (*Agaricus bisporus*) is 1-octen-3-ol [7], which is synthesized largely in the mushroom’s cap and gills [8], and in much higher amounts than in other edible mushrooms [9]. There is a very low olfactory threshold for 1-octen-3-ol [6], and thus, it is applied as a food additive authorized by the US Food and Drug Administration (ASP 1154, Regnum 172.515) [10]. In addition, 1-octen-3-ol also influences the growth, development, and behavior of fungi, plants, and insects (Kües, 2018; Holighaus, 2019) [11,12,13] as the signaling molecule oxylipin [14] and a quorum-sensing molecule [15]. In particular, 1-octen-3-ol is proposed to be a self-inhibitor for the primordium formation of the button mushroom [16,17].

The 1-octen-3-ol produced by the natural oxidation of linoleic acid or chemical synthetization is a mixture of two enantiomers of (R)-(−)-1-octen-3-ol and (S)-(+)-1-octen-3-ol in equal amounts [18], while (R)-(−)-1-octen-3-ol is predominantly produced by biosynthesis, especially in button mushrooms (up to 99% of (R)-(−)-1-octen-3-ol) [10]. (S)-(+)-1-octen-3-ol has a moldy, grassy aroma [19] and low biological activity [20]. Therefore, the application of the homogenates of button mushrooms to break down polyunsaturated fatty acids (PUFAs) such as linoleic acid for the production of 1-octen-3-ol has been commercialized [9,21,22]. However, the process conditions are very demanding, with the homogenization of the mushrooms occurring at 4 °C and enzyme reactions at 4–7.5 °C to avoid enzyme degradation by self-proteases. This is essentially due to the fact that the enzymes of *A. bisporus* for the synthesis of 1-octen-3-ol remain obscure. Once the enzymes of *A. bisporus* involved in the biosynthesis of 1-octen-3-ol are clearly identified, the enzymes can be exogenously expressed for large-scale applications. In addition, the biological functions of 1-octen-3-ol within the mushroom can be clarified.

The enzymes that cleave linoleic acid to generate oxylipins in fungi include mono-oxygenases, dioxygenases, and hydroperoxide lyases [14,23]. Monooxygenases introduce a single oxygen atom into the linoleic acid to mediate ω-oxidation [14]. Lipoxygenases (LOXs) are nonheme-containing dioxygenases that introduce molecular oxygen into linoleic acid to generate hydroperoxides containing conjugated dienes. Heme-containing dioxygenases (DOXs) insert molecular oxygen into linoleic acid to form hydroperoxides without conjugated dienes [24]. Fatty acid hydroperoxide lyase (HPL) catalyzes the cleavage of C–C bonds in the hydroperoxides of PUFAs to form short-chain aldehydes and ω-oxoacids [25].

The structure and function of fungal DOXs are quite diverse. Some DOXs contain only a fatty acid heme peroxidase/dioxygenase domain at the N-terminal, named DOX. Some DOXs are fusion enzymes containing a fatty acid heme peroxidase/dioxygenase domain at the N-terminus and a cytochrome P450 heme-thiolate domain at the C-terminus, cataloged as DOX-CYP. The type of DOX-CYP that has C-terminal CYP domains and linoleate diol synthase activity is designated as DOX-LDS or LDS. The type of DOX-CYP that has C-terminal CYP domains and epoxy alcohol synthase activity is designated as DOX-EAS. The type of DOX-CYP that has C-terminal CYP domains and allene oxide synthase activity is designated as DOX-AOS. The fusion enzymes with no functional P450 heme-thiolate domains are designated as DOX-(CYP) [14,23,26].

Several oxo- and hydroxy acids were found to produce 1-octene-3-ol in a reaction system of mushroom homogenates of *A. campestris* and linoleic acid. The *A. campestris* biochemical synthesis pathway of 1-octen-3-ol suggests that linoleic acid is first oxidized by LOX to form 13-hydroperoxide (13-HPOD), and 13-HPOD is cleaved by HPL into 1-octen-3-one and 10-oxodecanoic acid (10-ODA). Then, 1-octene-3-one is reduced by oxidoreductase to 1-octene-3-ol [27]. However, the protein extract of button mushrooms cleaved only 10-HPOD, not 13-HPOD, to generate 1-octene-3-ol and 10-ODA [3,28]. Further, 10-HPOD, not 13-HPOD, is the precursor of 1-octen-3-ol that is also observed in *Pleurotus pulmonurius* [29,30]. The stereochemical and volumetric correlations between 1-octen-3-ol and 10-HPOD have been confirmed in both *Lentinula edodes* and *T. matsutake* mushrooms [31]. Importantly, 10-HPOD does not have a conjugated diene motif and thus is not a LOX product. Furthermore, the specific inhibitor of DOX inhibits the synthesis of 1-octen-3-ol and 3-octanone but does not inhibit the synthesis of 1-octene-3-one in the reaction system of button mushroom homogenate and linoleic acid, indicating that DOX, not LOX, is involved in the synthesis of 1-octen-3-ol for *A. bisporus* [32].

*Aspergillus nidulans* is the first specimen in which the biosynthesis pathway of 1-octen-3-ol in fungi was identified. The *ppoC* gene of *A. nidulans* encodes a DOX-(CYP), which catalyzes the production of 10-HPOD from linoleic acid. Following this, 10-HPOD then decomposes into 10-ODA and 1-octene-3-ol [33]. Subsequently, this pathway was also found in *A. luchuensis* [34,35]. Alternatively, the LOX and HPL from *T. matsutake* co-expressed by yeast can catalyze the conversion of linoleic acid to produce 1-octene-3-ol [18]. However, the conserved domains in this LOX show that it is actually DOX-(CYP), and the precursor of 1-octene-3-ol was also not determined. The HPL from *T. matsutake* contains a C-22 sterol desaturase subfamily domain encoded by fungal cytochrome P450 61 (CYP61) or a related plant cytochrome P450 710 (CYP710), similar to the yeast C-22 sterol desaturase ERG5, but this domain does not belong to the plant HPL encoded by CYP74. Plant HPL characteristically cleaves 9-HPOD and/or 13-HPOD and does not form 1-octen-3-ol [36].

In the cyanobacterium *Nostoc punctiforme,* a dioxygenase and catalase act as linoleate 10*S*-dioxygenase and 10*S*-hydroperoxide lyase for the oxidative cleavage of linoleic acid to 1-octen-3-ol [37]. In seaweed and moss, a multifunctional lipoxygenase is responsible for the biosynthesis of 1-octen-3-ol from the oxidative cleavage of linoleic acid [38,39,40]. In soybeans, 1-octen-3-ol is formed by the degradation of unsaturated fatty acid derivative glycosides [41,42]. Thus, the biosynthesis pathways for 1-octen-3-ol are very diverse.

In this study, we retrieved four genes from the *A. bisporus* genome that may be involved in the oxidation and cleavage of linoleic acid to form 1-octene-3-ol. We cloned these genes and expressed them in prokaryotic systems. The products of the expressed enzymes that acted on linoleic acid were determined. The enzymatic pathway of *A. bisporus* for the biosynthesis of 1-octene-3-ol was demonstrated.

## 2. Materials and Methods

### 2.1. Strains and Plasmids

*A. bisporus* As2796 was obtained from the Edible Fungi Research Institute of the Fujian Academy of Agricultural Sciences (Fuzhou, China). The pBHG-BCA1-gpd plasmid was kindly gifted to us by Professor Baogui Xie from the Fujian Agriculture and Forestry University. The resistance marker hygromycin B phosphotransferase (hph) gene resided in the plasmid [43]. *E. coli* BL21 and plasmid pCold-TF were purchased from Zomanbio (Beijing, China).

### 2.2. Bioinformatics Analysis

Two LDS genes were retrieved from the *A. bisporus* var bisporus (H97) genome (https://mycocosm.jgi.doe.gov/Agabi_varbisH97_2/Agabi_varbisH97_2.home.html (accessed on 3 January 2020)) [44], named *AbLDS1* (NCBI accession number XP_006456120) and *AbLDS2* (NCBI accession number XP_006461894), while no *LOX*, *HPL*, or *CYP74* genes were retrieved. The sesquiterpene synthase gene *Cop4* of *Coprinus cinerea* and the three sesquiterpene synthase genes of *Hericium erinaceus* all encode linoleate 10R-lipoxygenase [45]. A Cop4 homologous protein was retrieved from the button mushroom genome and designated *Ab*LOX (NCBI accession number XP_006459300). A matsutake HPL homologous protein was retrieved from the button mushroom genome and designated as *Ab*HPL (NCBI accession number XP_006454582). The modular architectures of enzyme protein sequences were distinguished by using the Conserved Domain Search of NCBI. The multiple alignment of enzyme protein sequences was conducted using the ClustalW program (Dublin, Ireland).

### 2.3. Gene Cloning, Expression, and Purification

*A. bisporus* As2796 mycelia cultured in nutrient-rich potato dextrose agar medium [46] were collected. Total RNA was extracted from the mycelia by using an RNAiso Plus kit (Code No. 9108; Takara, Shiga, Japan) following the manufacturer’s instructions. cDNA synthesis from total RNA was performed using the RevertAid First cDNA Synthesis Kit (Thermo Scientific, Fair Lawn, NJ, USA). The four putative genes involved in the biosynthesis of 1-octene-3-ol in *A. bisporus* were cloned by PCR as follows. The reaction system comprised a 100 ng template cDNA, 1 μL primer (10 μM), 10 μL 2 × pfu PCR Master Mix (GenStar, Beijing, China), and ddH_2_O up to 20 μL. The primers used are listed in Table S1. The PCR was run at 94 °C for 5 min, 32 cycles of 94 °C for 30 s, 58 °C for 30 s, and 72 °C for 2 min followed by a 72 °C extension for 10 min. The PCR products were purified by using a DiaSpin DNA Gel Extraction Kit (Sangon Biotech, Shanghai, China) and sequenced by Henan Shangya Biotech (Zhengzhou, China).

The sequences of the PCR products were codon-optimized for *E. coli* expression using the genscript online software. The optimized sequences were synthesized by Shangya Biotech and ligated with the prokaryotic expression vector pET28a. The generated vectors were transformed into *E. coli* BL21 and expressed under the following conditions. When the cells grew to OD_600_ = 0.4–0.6 in LB liquid medium at 37 °C, IPTG was added to a final concentration of 0.4–0.6 mM, and then the cells were cultivated at 16 °C and 180 rpm for 12 h. The cells were collected and disrupted by using ultrasonic waves at 4 °C. The supernatant of the cell-disrupted solution was collected by centrifugation at 5000 *g* for 5 min at 4 °C and purified by using a nickel-ion affinity chromatography column (Sangon Biotech, Shanghai, China). The concentration of the purified protein was measured by using a Pierce^®^ BCA Protein Assay Kit (catalog number: 23225; Waltham, MA, USA).

### 2.4. Dioxygenase Activity Assay

LOX and LDS activity assays were performed by using the potassium iodine (KI)-starch method as described elsewhere [47]. Briefly, the linoleic acid solution (pH 6.5) was prepared according to the method previously described [48,49]. A mixture of 1 mL of enzyme extract, 1.5 mL of 0.1 M KH_2_PO_4_ buffer solution (pH 7.0), and 0.5 mL of 10 mM linoleic acid was incubated at 25 °C for 20 min. Subsequently, 0.03 mL of glacial acetic acid, freshly prepared saturated KI solution (0.4 mL), and 1% starch solution (0.1 mL) were added and mixed thoroughly. Then, the absorbance at 470 nm was determined for 8 consecutive min. The increase in absorbance of 0.001 within 1 min was defined as one enzyme activity unit.

### 2.5. HPL Activity Assay

An aliquot of 0.5 mL of 10 mM linoleic acid solution (pH 6.5) mixed with 0.01 mL of 0.01 M of hydrogen peroxide solution was incubated at 25 °C for 10 min to prepare the substrate fatty acid hydroperoxide. Then, 0.5 mL of *Ab*HPL extract was added, and the reaction system was made up to 3 mL using 0.1 M PBS buffer. The reaction was continued at 25 °C for 30 min. Subsequently, 20 μL of glacial acetic acid, 1 mL of deionized water, and freshly prepared saturated KI solution (0.4 mL) and 1% starch solution (0.1 mL) were added and mixed thoroughly. Soon afterward, the absorbance at 470 nm was measured for 8 consecutive min. One enzyme activity unit was defined as the amount of enzyme that caused a decrease in absorbance of 0.001 per min.

### 2.6. Enzymatic Synthesis of 1-octene-3-ol from Linoleic Acid

To a glass bottle, 1 mL of 15 mM linoleic acid (pH 6.5), 0.5 mL of *Ab*LOX, *Ab*LDS1 or *Ab*LDS2 extract, and 0.5 mL of *Ab*HPL extract were added. The bottle was sealed and incubated at 25 °C for 1 h.

### 2.7. Headspace Solid-Phase Micro-Extraction Gas Chromatography–Mass Spectrometry (HS-SPME-GC–MS) Analysis

The products of the enzymatic synthesis of 1-octene-3-ol from linoleic acid were extracted with an equal volume of ether for 30 min. The supernatant was carefully collected after centrifugation at 5000× *g* for 8 min. Then, the supernatant was evaporated in a fume hood until the volume was concentrated to approximately 1 mL. The volatiles were enriched by using HS-SPME (50/30 μm DVB/CAR/PDMS spme, Supelco, Bellefonte, PA, USA). A GC Trace1300 gas chromatograph coupled with ISQ-Mass (Thermo Scientific, Fair Lawn, NJ, USA) was used for volatile profiling. After enrichment, the SPME assembly was evacuated from the bottle and plugged into the GC injection port, where it remained for 20 min at 220 °C for volatile desorption. The separation was attained within a DB-5MS column (30 m × 0.25 mm × 0.25 μm). The carrier gas was high-purity helium with a flow rate of 1.0 mL/min. The split mode was 2:1. The injector temperature was 250 °C. The oven temperature program ranged from 50 °C to 280 °C at a ramp rate of 10 °C/min. The MS data were received by the mass detector operating at full scan in the range of 20–500 amu.

The production of 1-octen-3-ol by the plate mycelia was also detected by HS-SPME-GC–MS. The surface mycelia were collected after the plates were cultivated for 15 d. A sample of 0.02 g of mycelia was placed into a 5 mL sampling bottle and incubated in a 50 °C water bath for 30 min to prepare the gas for measuring.

### 2.8. Construction of Antisense AbLOX Transformants

Antisense *AbLOX* (as*AbLOX*)-containing homology arm sequences were obtained by PCR using *A. bisporus* cDNA as a template with the primer pairs HRasAbLOX-F and HRasAbLOX-R (Table S1). Subsequently, as*AbLOX* was ligated with the linearized plasmid pBHG-BCA1-gpd by homologous recombination using an SE Seamless Cloning and Assembly Kit (ZC231, Zomanbio, Beijing, China), resulting in the as*AbLOX* expression vector pBHG-asAbLOX-gpd. pBHG-asAbLOX-gpd was transformed into *A. bisporus* using the Agrobacterium-mediated fruit body tissue culture transformation method [50]. The as*AbLOX* transformants were verified by the PCR detection of the hygromycin resistance gene and as*AbLOX* gene. The primer pairs used were Hyg-F and Hyg-R, and asAbLOX-F and asAbLOX-R (Table S1).

### 2.9. Quantitative RT–PCR

The expression of *AbLOX* in *A. bisporus* mycelia was tested by quantitative RT–PCR as previously described [46]. The primer pairs used were RT-LOX-F and RT-LOX-R, and Ef1a-F and Ef1a-R (Table S1); the reference gene was Ef1a. Each 20 µL reaction contained 10.0 µL of ChamQ Universal SYBR qPCR Master Mix (2×), 0.4 µL of primer (10 µM), and 100 ng of cDNA derived from the total RNA extracted from mushroom mycelia. The thermal cycling profile was 95 °C for 30 s, followed by 40 cycles at 95 °C for 5 s and 60 °C for 20 s. The gene expression compared to the wild type was calculated using the 2^−ΔΔCt^ method.

### 2.10. Statistical Analysis

The results are expressed as the mean values or percentages ± standard error (n = 3) of three independent experiments. The statistical analysis was conducted by using SPSS software package (Version 13.0, SPSS, Chicago, IL, USA).

## 3. Results

### 3.1. Homology Analysis of 1-octen-3-ol Synthesis-Related Genes in Agaricus bisporus

To analyze the enzymatic pathway of *A. bisporus* for the biosynthesis of 1-octen-3-ol, four putative genes related to the synthesis of 1-octen-3-ol were acquired from the *A. bisporus* H97 genome by a homology search. The deduced amino acid sequence for *AbLOX* contained a terpene_syn_C_2 domain, which was 62.83% identical to the linoleate 10R-lipoxygenase Cop4 from *C. cinerea* (Figure S1), suggesting that *AbLOX* may be a lipoxygenase gene. The proteins encoded by *AbLDS1* and *AbLDS2* both contained DOX and CYP domains. Their CYP domains were similar to those of the PpoC of *A. nidulans* [33], the PpoC of *A. luchuensis* [34], and the LOX1 of matsutake [18] with no intact conserved heme-binding region, and were therefore nonfunctional. In contrast, the CYP domain of *A. nidulans* PpoA had an intact conserved heme-binding region and was functional [33] (Figure S2). The deduced amino acid sequence of *Ab*HPL had the same cytochrome P450 superfamily domain structure as the HPL of matsutake mushroom with known functions, and their amino acid sequence homology reached 65.10% (Figure S3). Thus, *Ab*HPL probably had HPL activity.

### 3.2. Enzyme Activity of Prokaryotic-Expression Proteins

To validate the functions of the four putative genes of *A. bisporus* related to the synthesis of 1-octen-3-ol, the four genes were cloned by PCR from *A. bisporus* As2796 using primers designed from the genome sequence of *A. bisporus* H97. The sequences of the four genes of *A. bisporus* As2796 were completely identical to those of *A. bisporus* H97. The four enzymes encoded by the genes were expressed in *E. coli* and purified by an affinity chromatography column (Figure S4). The *Ab*LOX, *Ab*LDS1, and *Ab*LDS2 extracts were all capable of catalyzing the conversion of linoleic acid to hydroperoxide, while the *Ab*HPL extract was capable of catalyzing the reduction in fatty acid hydroperoxide. The specific activities of the four enzymes tested by the KI-starch method were 8.1–67.5 U/mg (Table 1).

### 3.3. Analysis of the Volatile Products of Enzymatic Linoleic Acid Degradation by HS-SPME-GC–MS

**Figure 1:**
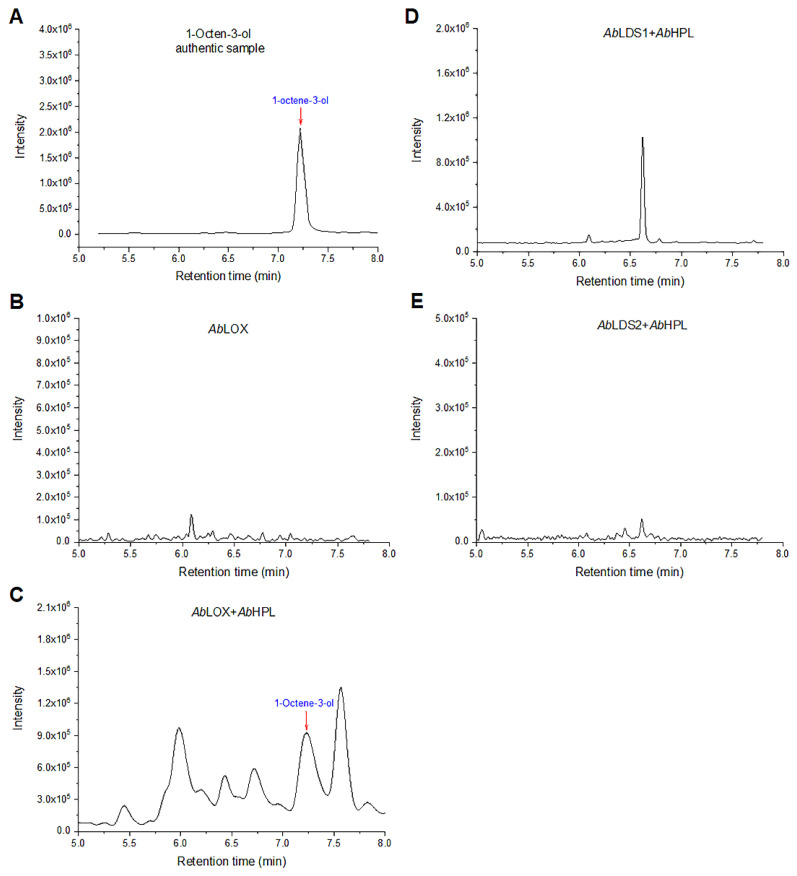
GC-MC analysis chromatogram of 1-octen-3-ol production in the enzymatic linoleic acid reaction. (**A**) Authentic sample of 1-octen-3-ol. (**B**) Enzymatic reaction by *Ab*LOX. (**C**) Enzymatic reaction by *Ab*LOX + *Ab*HPL. (**D**) Enzymatic reaction by *Ab*LDS1 + *Ab*HPL. (**E**) Enzymatic reaction by *Ab*LDS2 + *Ab*HPL.

To examine whether the putative enzymes involved in the synthesis of 1-octen-3-ol catalyze the production of 1-octen-3-ol from linoleic acid, the *Ab*LOX, *Ab*LDS1, and *Ab*LDS2 extracts were each incubated with *Ab*HPL extract and linoleic acid, and then their volatile products were analyzed using HS-SPME-GC–MS. The peak and mass spectrum of 1-octen-3-ol were detected only in the *Ab*LOX + *Ab*HPL reaction at the same retention time (RT = 7.28 min) as that of the 1-octen-3-ol standard, but this same peak was not detected in the reactions of *Ab*LDS1 or *Ab*LDS2 with *Ab*HPL and *Ab*LOX alone (Figure 1 and Figure S5).

### 3.4. The Optimal Temperature and pH for the Enzymatic Synthesis of 1-octen-3-ol from Linoleic Acid

**Figure 2:**
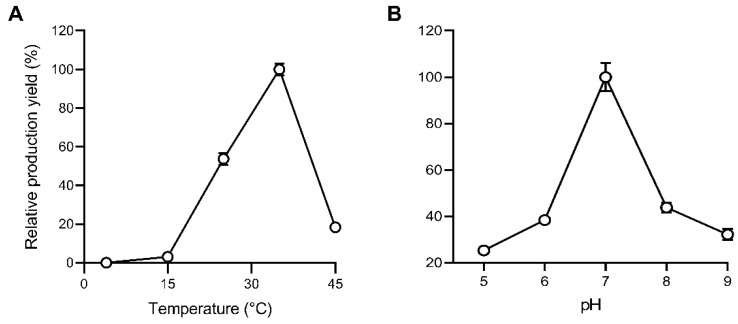
Effects of temperature (**A**) and pH (**B**) on the production of 1-octen-3-ol from linoleic acid by *Ab*LOX and *Ab*HPL.

For the effective production of 1-octen-3-ol by *Ab*LOX and *Ab*HPL, the optimal temperature and pH for the enzymatic reaction were studied. The optimal reaction temperature was studied by detecting the enzymatic reaction production of 1-octen-3-ol by SPME-GC after the reaction for 1 h over a temperature range of 4–45 °C and pH 7.2 with the substrate 3 mM linoleic acid. Briefly, 1-octen-3-ol was not detected as a product for the reaction conducted at 4 °C. The optimal reaction temperature was 35 °C, whereas the relative production yield sharply declined at 45 °C (Figure 2A). The optimal reaction pH was studied over a pH range of 5.0–9.0 at 35 °C with a substrate of 3 mM linoleic acid for 1 h. The optimal reaction pH was 7.0, which correlated with the maximum production yield (Figure 2B).

### 3.5. Effect of Reduced AbLOX Expression on Agaricus bisporus Mycelial Growth and the Production of 1-octen-3-ol

**Figure 3:**
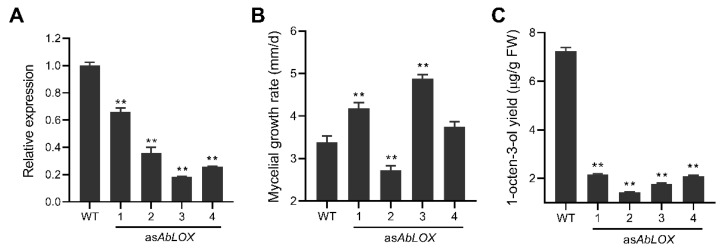
The *Ab*LOX expression (**A**), mycelial growth rate (**B**) and mycelial 1-octen-3-ol yield (**C**) of the antisense *Ab*LOX transformants derived from *Agaricus bisporus*. WT, The parent strain of *A*. *bisporus* As2796; as*AbLOX*-1 to *asAbLOX*-4, The antisense *AbLOX* transformants. Data marked with (**) were significantly different at *p* < 0.01 from WT based on one-way ANOVA.

To confirm that *AbLOX* was also involved in the synthesis of 1-octen-3-ol in vivo, as*AbLOX* transformants of *A. bisporus* were constructed. After five generations of subculture, four transformants were obtained and named *AbLOX*-1 to *AbLOX*-4. The mycelial growth rate of the four transformants in nutrient-rich potato dextrose agar medium was not lower than that of the parent, except for *AbLOX*-2 (Figure 3A). The expression of the *AbLOX* in the four transformants was 34–82% lower than that of the parent (Figure 3B), and the production of 1-octen-3-ol was 70–80% lower than that of the parent (Figure 3C). In addition, the *AbLOX* expression of all strains showed a significant positive correlation with their 1-octene-3-ol yield (*p* < 0.01).

## 4. Discussion

Several studies have confirmed that DOXs oxidize linoleic acid to 10-HPOD, and HPLs specifically cleave 10-HPOD to 1-octen-3-ol and 10-ODA in *A. bisporus* [3,28,32]. However, to date, the corresponding enzymes have not been identified. In this study, we validated the idea that the combination of heterologously expressed *Ab*LOX and *Ab*HPL catalyzed the oxidative cleavage of linoleic acid to 1-octen-3-ol with an optimum pH of 7.2, in agreement with the crude enzyme homogenate obtained from the mushrooms [21]. A reduced *AbLOX* expression correlated with the decreased production of 1-octen-3-ol by *A. bisporus* mycelia, suggesting that *Ab*LOX may be the only fatty acid oxidase that catalyzes the production of 1-octen-3-ol from linoleic acid in *A. bisporus*, most likely as DOX. In addition, the reaction catalyzed by *Ab*LOX was probably the rate-limiting step in the synthesis of 1-octen-3-ol from linoleic acid in *A. bisporus*.

*Ab*LOX was highly homologous to the sesquiterpene synthase Cop4 of *C*. *cinerea*. Interestingly, a dioxygenase from *P. sapidus* was able to convert the sesquiterpene (+)-valencene to (+)-nootkatone, as well as convert linoleic acid to 13-HPOD; since it is functional as a lipoxygenase, it was designated as LOXPsa1 [51]. In addition, LOXPsa1 can oxidize piperine to piperonal and 3,4-methylenedioxycinnamaldehyde depending on the co-oxidation of linoleic acid [52]. The multisubstrate property of sesquiterpene synthase from fungi has not yet been reported. This study found that *Ab*LOX was capable of catalyzing the formation of fatty acid hydroperoxides from linoleic acid, possibly with multiple substrate properties.

Plant HPLs are CYP74 proteins that catalyze the cleavage of 9- and/or 13-HPODs to form an aldehyde and ω-oxo-acid [36]. The HPL from the cyanobacterium *Nostoc punctiforme* is a heme catalase with 10-HPOD cleavage activity [37], whereas the other bacterial HPL from *Methylobacterium nodulans* is a CYP74 protein with 13-HPOD cleavage activity (53). A small number of identified HPLs from animals were the CYP74 clan proteins [53,54,55]. An HPL cloned from matsutake was considered to be capable of cleaving HPOD to form 1-octen-3-ol [18], which was highly homologous to sterol C-22 desaturase encoded by CYP61 in *Saccharomyces cerevisiae* [56]. Whether sterol C-22 desaturase has multisubstrate properties was not recorded. In this study, *Ab*HPL, with a high homology compared to HPL from matsutake, can catalyze the products of *Ab*LOX oxidation of linoleic acid to generate 1-octen-3-ol, indicating that *Ab*HPL has different structures and functions to HPLs in plants, animals, and bacteria.

A large quantity of 1-octen-3-ol was synthesized by *A. bisporus* mycelia and its developed fruiting bodies [32,57,58,59]. Similarly, *C. cinerea* mycelia and its developed fruiting bodies also yielded high quantities of 1-octen-3-ol as well as sesquiterpenes, such as β-himachalene and cuparene [60]. The LOX and CYP74 protein genes were not retrieved from the genome of *C. cinerea* okayama 7#130 [61], whereas six genes of terpenes synthase [62]; three genes of LDS, all of which were DOX-(CYP), and one gene of sterol C-22 desaturase were retrieved. The deduced amino acid sequence of the sterol C-22 desaturase was 69.85% identical to *Ab*HPL. Furthermore, six genes encoding terpene synthases, including *AbLOX,* were retrieved from the genome of *A. bisporus* H97 [44]. Therefore, *A. bisporus* and *C. cinerea* should have a similar biosynthesis pathway for 1-octen-3-ol. Nonetheless, the enzymatic pathways for the biosynthesis of 1-octen-3-ol in mushroom fungi were probably quite diverse. A high amount of 1-octen-3-ol was synthesized in the growing mycelia and early developing fruiting bodies of *Cyclocybe aegerita*. A total of two putative DOX-CYP genes, five putative LOX genes, and eleven putative HPL (CYP74 proteins) genes were found in the *C. aegerita* transcriptome. The course of expression of one putative DOX-CYP gene and one HPL gene in the mycelial stages and fruiting body samples showed similarities to the 1-octen-3-ol pattern, suggesting that the two genes are involved in the biosynthesis of 1-octen-3-ol [63]. The production of 1-octen-3-ol in the postharvest fruiting bodies of *L. edodes* continued to decrease. Five LOX-related genes were identified from the transcriptomes in the postharvest fruiting bodies of *L. edodes*, the expression levels of one in which were positively correlated with 1-octen-3-ol, indicating that one of the LOX genes was responsible for regulating the biosynthesis of 1-octen-3-ol [64].

## 5. Conclusions

In summary, the enzymes that cleaved linoleic acid to 1-octen-3-ol in *A. bisporus* were not the common enzymes for the synthesis of oxylipids but multifunctional fatty acid dioxygenase *Ab*LOX and hydroperoxide lyase *Ab*HPL, and this may be the reason why the enzymatic pathway for the biosynthesis of 1-octen-3-ol in *A. bisporus* has not been elucidated until now. The enzymatic pathway for the biosynthesis of 1-octen-3-ol in *A. bisporus* discovered in this study will considerably contribute to the in-depth exploration of the biological functions of 1-octen-3-ol and oxylipids for mushroom fungi and the efficient production of the natural isomers of 1-octen-3-ol.

## Figures and Tables

**Table 1:** Activities of the prokaryotic-expressed enzymes related to the synthesis of 1-octen-3-ol by *Agaricus bisporus*.

Enzyme	Specific Activity (U/mg of Protein)
*Ab*LOX	55.9 ± 2.4
*Ab*LDS1	51.7 ± 1.5
*Ab*LDS2	67.5 ± 3.0
*Ab*HPL	8.1 ± 0.1

## Data Availability

The datasets are available from the corresponding author on reasonable request.

## Supplementary Materials

The following supporting information can be downloaded at: https://www.mdpi.com/article/10.3390/jof8080827/s1, Figure S1: The amino acid sequence identify between *Ab*LOX in *Agaricus bisporus* H97 and Cop4 in *Coprinus cinereus*. Figure S2: Domain structure and catalytic active site of the putative heme dioxygenase from *Agaricus bisporus* H97 and other selected fungi. Figure S3: The amino acid sequence identified between *Ab*HPL in *Agaricus bisporus* H97 and *Tm*HPL in *Tricholoma matsutake*. Figure S4. Protein purification of the putative enzymes involved in 1-octen-3-ol biosynthesis by nickel ion affinity chromatography column. Figure S5. Mass spectrum of 1-octen-3-ol from the enzymatic linoleic acid reaction by AbLOX+AbHPL. Table S1: Primers used in this study.
