# Supplementary material for: The Biosynthesis of 1-octene-3-ol by a Multifunctional Fatty Acid Dioxygenase and Hydroperoxide Lyase in Agaricus bisporus
Source: J Fungi (Basel). 2022 Aug 8;8(8):827. doi: 10.3390/jof8080827 (PMC9410191; doi:10.3390/jof8080827)
Supplement: Supplementary file 1 [file jof-08-00827-s001.zip › jof-1824520-supplementary.pdf]

SUPPLEMENTARY MATERIAL

Table S1. Primers used in this study

Primer name	Sequence (5′–3′)[a]
AbLOX-F1	ATGCCTCGCCCTCAACAATTC
AbLOX-R1	CTCGCAATCTGAATCTGAGTCTACC
AbLOX-F	CATGCCATGGGC ATGCCTCGCCCTCAACAATTC
AbLOX-R	AAGGAAAAAAGCGGCCGCCTCGCAATCTGAATCTGAG
AbLDS1-F1	ATGTCCCACTTGACAAATCTAC
AbLDS1-OL-R	CCCATAACTTCAATCGCCTTC
AbLDS1-OL-F	GAAGGCGATTGAAGTTATGGG
AbLDS1-R1	TTACACTTTCACCTGCAACTG
AbLDS1-F	*GGGTACCCTCGAGGGATCCGAATTC*ATGTCCCACTTGACAAATCTACTTCT
AbLDS1-R	*TTTAAGCAGAGATTACCTATCTAGA*TTACACTTTCACCTGCAAC
AbLDS2-F1	ATGTCCAAGAGGCTCTCTGG
AbLDS2-OL-R	CAGGATGGCACGACTAATGG
AbLDS2-OL-F	CCATTAGTCGTGCCATCCTG
AbLDS2-R1	AGCATCATACTGAATGGTAAGAGAC
AbLDS2-F	*TGGTGGTATCGAAGGTAGGCATATG*ATGTCCAAGAGGCTCTCTGGTATC
AbLDS2-R	*TTTAAGCAGAGATTACCTATCTAGA*AGCATCATACTGAATGG
AbHPL-F1	ATGGTCCTTGACGTTCACGCT
AbHPL-R1	TCATGCTTGCGCACGAGG
AbHPL-F	GGAATTCCATATGGTCCTTGACGTTCACGCT
AbHPL-R	CCCGCGGCCGCTCATGCTTGCGCACGAGG
HRasAbLOX-F	*ACACAACAACTTATCACTAGT*CTCGCAATCTGAATCTGAGTCTACC
HRasAbLOX-R	*TGCATGCCAATTCTAGAGGGCCC*CCTCGCCCTCAACAATTCATT

Hyg-F	CTTCTGCGGGCGATTTGTG
Hyg-R	TCGTTATGTTTATCGGCACTTT
asAbLOX-F	CTCGCAATCTGAATCTGAGTCTACC
asAbLOX-R	CCTCGCCCTCAACAATTCATT
Ef1a-F	AACAAGGTTCCCCTGGTGAC
Ef1a-R	CGGGTGATTGAGGACGATG
RT-LOX-F	AGCTCCGAGCGACTGTTGGC
RT-LOX-R	GGGTGCGTTTGATCTCGTCA

[a] Italic letters show homology arm sequence

```
AbLOX   -MPRPQQFILPDLLSSCPLEDGLNPHYREAAAESRTWINSFNIFSNRKRADFIQGLNELL   59
Cop4    MRPTARQFTLPDLFSICPLQDATNPWYKQAAAESRAWINSYNIFTDRKRAFFIQGSNELL   60

AbLOX   CSHVYCYAGYEEFRTTCDFVNVLFVVDEISDEQSGKDARATGLSYAESMRNADWDDNSVV  119
Cop4    CSHVYAYAGYEQFRTCCDFVNLLFVVDEISDDQNGQDARATGRIFVNAMRDAHWDDGSIL  120

AbLOX   AKITKEFRARLIRRAGHNNFRRFVASSDAYTRCVGREAELREAGEVLSLEEYIPLRRNNS  179
Cop4    AKITHEFRERFVRLAGPKTVRRFADLCESYTDCVAREAELRERNQVLGLNDFIALRRQNS  180

AbLOX   AVLLCFDLVEYILGVDLPESIYQNATFLKAYWAACDHVCWCNDVYSYNVEQSKGHTGNNV  239
Cop4    AVLLCYSLVEYILGIDLDDEVYEDPTFAKAYWAACDFVCWANDVYSYDMEQAKGHTGNNV  240

AbLOX   VTVLMNDRQIGLQEACDYIGDRCRQFMNDYLAARDELRA--TVGGDASRFIDALGYWIIG  297
Cop4    VTVLMKEKDLSLQEASDYIGRECEKQMRDYLEAKSQLLQSTDLPQEAVRYIEALGYWMVG  300

AbLOX   NMEWSFESPRYFGHEHDEIKRTLTLTLKPSEVPEE---VDSDSDCE          340
Cop4    NLVWSFESQRYFGAQHERVKATHVVHLRPSSVLEASCDSDSDSDC-          345
```

Figure S1. The amino acid sequence identify between *Ab*LOX in *Agaricus bisporus* H97 and Cop4 in *Coprinus cinereus*. *Ab*LOX, putative lipoxygenase in *A. bisporus* H97 (NCBI accession number XP_006459300); Cop4, linoleate 10R-lipoxygenase Cop4 (NCBI accession number XP_001836356).

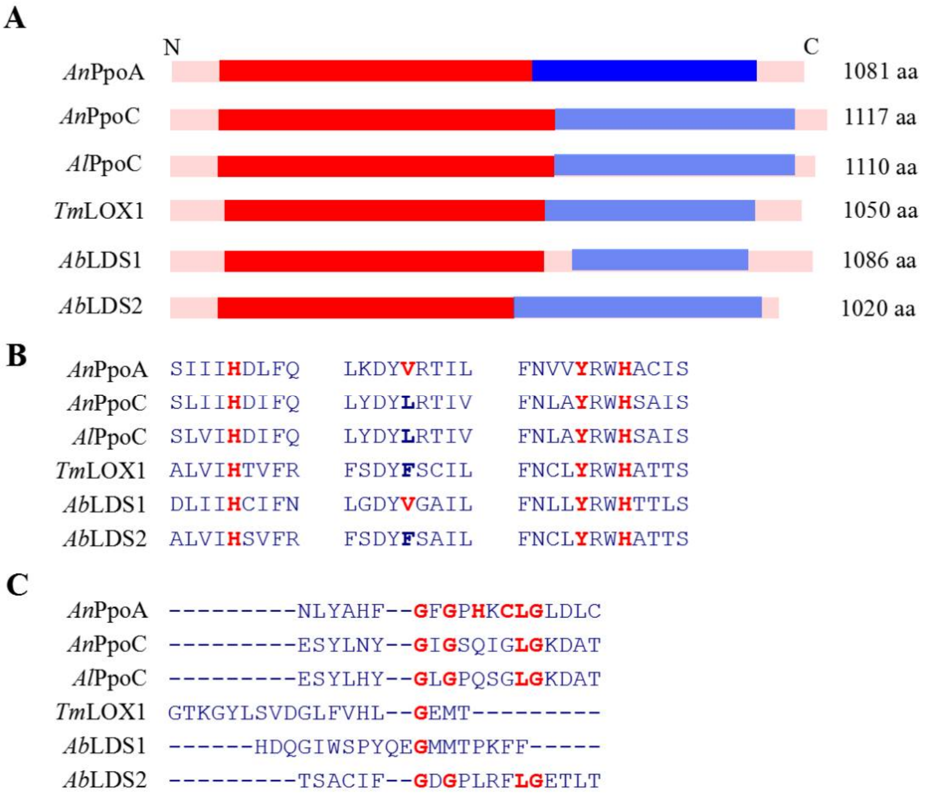

Figure S2. Domain structure and catalytic active site of the putative heme dioxygenase from *Agaricus bisporus* H97 and other selected fungi. A, Domain structure. Solid red box shows the heme peroxidase/dioxygenase domain; solid blue box shows the heme-thiolate domain with isomerization activity; solid light blue box shows the heme-thiolate domain without isomerization activity. B, Bold red letters show the conserved amino acid residues in the active site, bold purple letters show non-conserved amino acid residues in the active site. C, Heme-thiolate domain amino acid sequence alignment. Bold red letters show amino acid residues that are conserved in the catalytically active site. *Ab*LDS1, linoleate diol synthase 1 in *A. bisporus* H97 (NCBI accession number XP_006456120); *Ab*LDS2, linoleate diol synthase 2 in *A. bisporus* H97 (NCBI accession number XP_006461894); *An*PpoA, *Aspergillus nidulans* PpoA

(NCBI accession number AAR88626); *An*PpoC, *As. nidulans* PpoC (NCBI accession number AAT36614); *Al*PpoC, *As. luchuensis* PpoC (NCBI accession number GAT23542); *Tm*LOX1, *Tricholoma matsutake* lipoxygenase 1 (NCBI accession number KAF8239383).

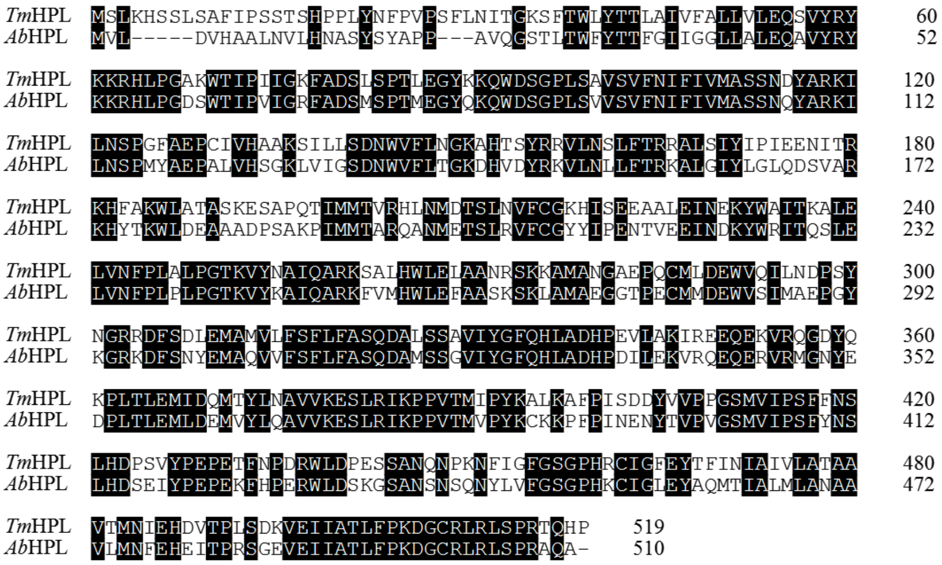

Figure S3. The amino acid sequence identify between *Ab*HPL in *Agaricus bisporus* H97 and *Tm*HPL in *Tricholoma matsutake*. *Ab*HPL, putative fatty acid hydroperoxide lyase in *A. bisporus* H97 (NCBI accession number XP_006454582); *Tm*HPL, fatty acid hydroperoxide lyase in *T. matsutake* (NCBI accession number KAF8221779).

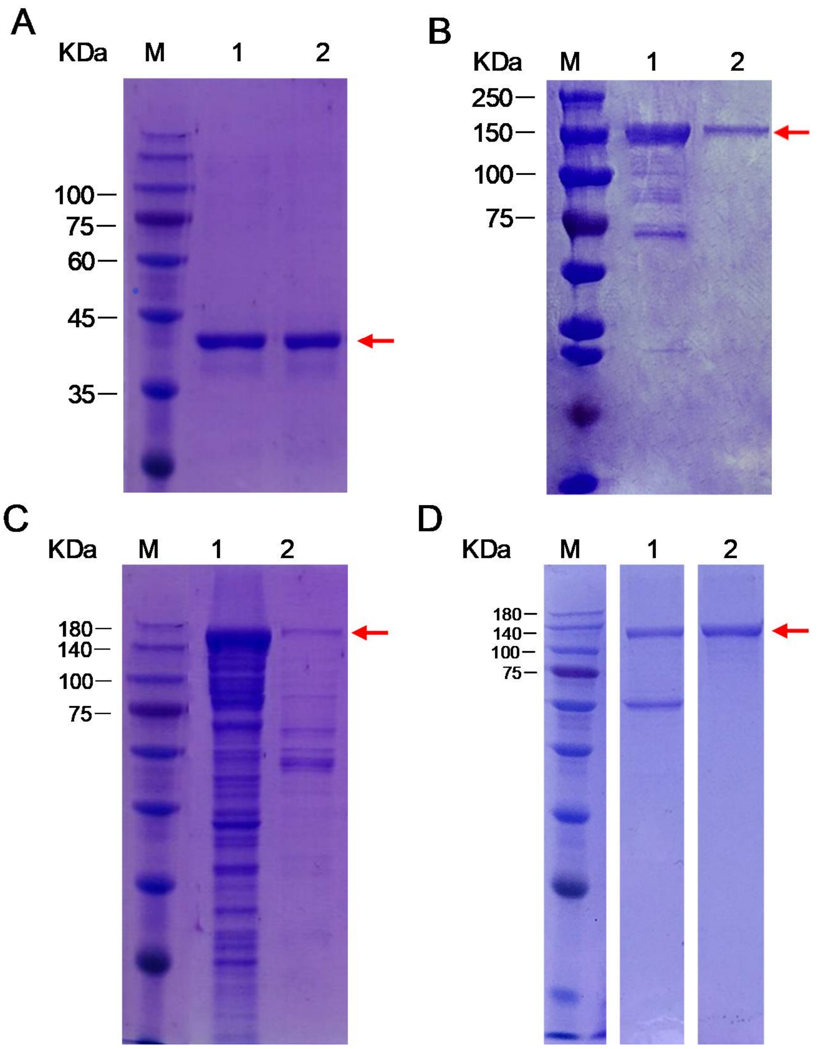

Figure S4. Protein purification of the putative enzymes involved in 1-octen-3-ol biosynthesis by nickel ion affinity chromatography column. (A) *Ab*LOX. Lane 1 and 2, elution with 100 and 150 mM imidazole. (B) *Ab*LDS1. Lane 1 and 2, elution with 100 and 150 mM imidazole. (C) *Ab*LDS2. Lane 1 and 2, elution with 20 and 100 mM imidazole. (D) *Ab*HPL. Lane 1 and 2, elution with 100 and 150 mM imidazole.

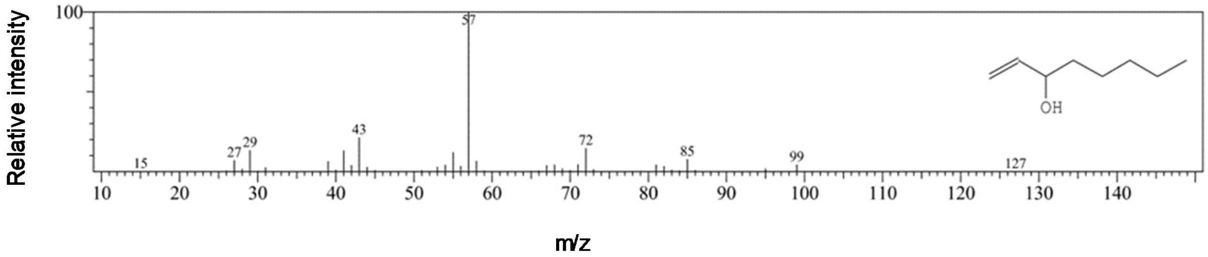

Figure S5 Mass spectrum of 1-octen-3-ol from the enzymatic linoleic acid reaction by *Ab*LOX+*Ab*HPL
